# Narcotic Requirements before and after Implementation of Buccal Nerve Blocks for Buccal Mucosa Graft Harvest: Technique and Retrospective Review

**DOI:** 10.3390/jcm12062168

**Published:** 2023-03-10

**Authors:** Adam Nolte, Alejandra Perez, Chase Mallory, Timothy Demus, Jessica Boyer, Scott Jamieson, Dhaval Jivanji, Billy Cordon

**Affiliations:** 1 Department of Urology, Mount Sinai Medical Center, Miami, FL 33140, USA; 2 Department of Urology, University of Florida, Gainesville, FL 32610, USA; 3 College of Osteopathic Medicine, Nova Southeastern University, Davie, FL 33328, USA; 4 School of Medicine, Florida International University, Miami, FL 33199, USA

**Keywords:** buccal mucosa graft, urethroplasty, opioid analgesia, nerve block

## Abstract

The reduction in opioid use has become a public health priority. We aimed to assess if performing buccal nerve blocks (BNB) at the time of buccal mucosa graft (BMG) harvest impacts post-operative narcotic usage in the inpatient setting. We retrospectively reviewed clinical characteristics and morphine milligram equivalents (MMEs) received for all patients that underwent a BMG urethroplasty at our institution. The primary outcome measure was post-operative MMEs for patients before and after implementing the BNB. We identified 74 patients that underwent BMG urethroplasty, 37 of which were before the implementation of the BNB and 37 of which were after. No other changes were made to the peri-operative pathway between these time points. The mean total MMEs per day, needed post-operatively, was lower in the BNB group (8.8 vs. 5.0, *p* = 0.12). A histogram distribution of the two groups, categorized by number of MMEs received, showed no significant differences between the two groups. In this retrospective analysis, we report our experience using BNBs at the time of buccal mucosa graft harvest. While there were no significant differences between the number of MMEs received before and after implementation, further research is needed to assess the blocks’ impact on pain scores.

## 1. Introduction

The opioid epidemic is a growing public health emergency, and physician over prescription of these medications is thought to be contributory. It is estimated that 1–13% of opioid-naïve patients progress to persistent use after surgery, even for minor urologic procedures [1,2,3,4]. Furthermore, more than 50% of opioid medications prescribed to post-surgical patients go unused, and can find their way into the community [1,5,6,7].

In response to the growing crisis, surgeons have been encouraged to pursue non-opioid pain management. One such approach involves the use of regional pre-operative nerve blocks and local anesthesia, which are thought to prevent the transition from acute to chronic neuropathic pain, and limit the pro-inflammatory response to surgery [8,9,10,11]. Nerve blocks have limited acute post-operative pain in a variety of urologic surgeries, including nephrectomy, percutaneous nephrolithotomy, and tunica plication [11,12,13,14,15,16,17].

Despite the growing use of nerve blocks for anesthesia, their use in BMG urethroplasty remains largely unstudied. A recent randomized trial demonstrated reduction in acute post-operative narcotic use following liposomal bupivacaine infiltration to the buccal mucosal harvest site, which is thought to be the foremost source of post-operative pain for these patients [18,19].

We hypothesized that adding the buccal nerve block would reduce pain, and thus reduce the number of opioid pain medications received by a patient. We reviewed the use of post-operative narcotics before and after this change, to assess its impact on narcotic requirements. Our study contributes to the literature by detailing how buccal nerve blocks can be performed at the time of buccal mucosa graft harvest. 

## 2. Materials and Methods

In an effort to improve the patient experience, control post-operative pain, and limit the use of post-operative narcotic medications, our institution began performing buccal nerve blocks (BNBs) intra-operatively for all BMG urethroplasties. This change of practice occurred in January 2019. We retrospectively reviewed clinical and procedural characteristics for all patients that underwent urethroplasty at our institution from August 2016 through February 2021. The study included all patients receiving a buccal mucosa graft harvest (BMG) as part of their treatment of urethral strictures. As part of our routine practice, all patients received general anesthesia. Moreover, patients throughout the study, in both groups, that had a perineal incision, received a pudendal nerve block.

### 2.1. Buccal Nerve Blocks 

Beginning in January 2019 we began performing BNB prior to BMG harvest. Blocks are performed by infiltrating 5 cc of a 50/50 mixture of 1% xylocaine and 25% bupivacaine with 4 mg of dexamethasone into the buccal sulcus just distal to the last molar tooth. We then proceed with the harvest. Our technique utilizes hydrodissection with both xylocaine and epinephrine. The harvest site is left open to heal by secondary intention. After harvesting, a gauze soaked with xylocaine and epinephrine is placed on the site and removed prior to leaving the operating room. 

### 2.2. Data Analysis

We divided our patient population into two groups, one before, and one after implementation of the BNB. The primary outcome was morphine milligram equivalents (MMEs), which is the amount of morphine equal to the strength of opioid prescribed. We looked at MMEs in the acute post-operative period, both in the post anesthesia care unit (PACU), and during the inpatient hospital stay. Additionally, we collected data regarding patient demographics, length of stay, graft size, incision location, and documented intra-operative stricture length. 

Due to the non-normal distribution of MMEs in our data set, we used non-parametric (Mann–Whitney U) testing to detect differences between MMEs given to each group in various phases of post-operative care. We also grouped MMEs to generate a histogram, and performed a Chi-Square analysis to detect differences between these groups. Analysis was conducted in Microsoft Excel. 

## 3. Results

Seventy-four patients underwent urethroplasty with buccal mucosa graft (BMG) harvest at our institution during the study period. Thirty-seven patients had surgery prior to January 2019 and were categorized as “Non-BNB”, indicating they had their surgery before implementation of the buccal nerve block (BNB). The remaining 37 patients were thus in the “BNB” group and received a BNB prior to BMG harvest.

Patient demographic factors including age, length of stay, stricture length, graft size, incision location, race, and ethnicity are shown in Table 1. The mean ages were 61.5 ± 12.8 and 59.7 ± 15.4 years in the “Non-BNB” and “BNB” groups, respectively. The mean intra-operative stricture lengths were 5.64 ± 3.76 cm and 5.20 ± 2.66 cm in the “Non-BNB” and “BNB” groups, respectively. Length of stay was one day in the majority of cases (31/37 patients in the “Non-BNB” group and 25/37 for the “BNB” group). Incisions were most commonly isolated to the penile urethra (54.1% in the “Non-BNB” group and 48.6% in the “BNB” group).

**Figure 1:**
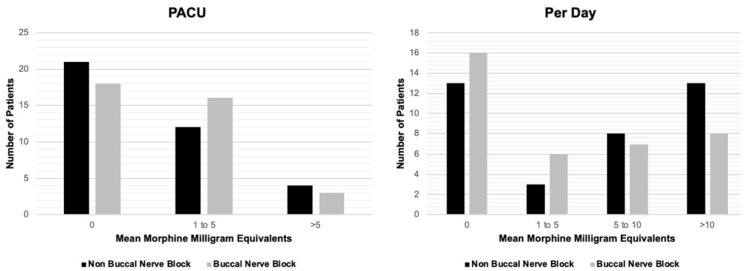
Histogram distributions for MMEs in Non-Buccal Nerve Block and Buccal Nerve Block groups stratified by phase of care (PACU *p* = 0.62 *, Per Day *p* = 0.46 *). * Chi-Square Test.

The mean and median MMEs received in PACU, per day, and throughout the hospital stay for each group are shown in Table 2. The total MMEs given decreased between the “Non-BNB” and “BNB” groups across all three groups, however, no groups reached statistical significance. Further analysis grouped patients (0 MMEs, 1–5 MMEs, etc.), however, the Chi-Squared test failed to show any significant differences between the groups. Results are shown in Figure 1. 

Finally, patient post-operative notes and outpatient records were analyzed for adverse reactions to the blocks. No discernable adverse effects were noted in the BNB group. 

## 4. Discussion

In an effort to control post-operative pain and limit the need for opioid medications in the post-operative period, we began performing a buccal nerve block (BNB) during buccal mucosa graft (BMG) harvests in January 2019. The buccal nerve is a branch of the mandibular nerve, which branches from the trigeminal nerve and is thought to innervate the buccinator muscles, cheek, gingival mucosa of the retromolar area, and buccal mucosa [20]. 

Prior experience with nerve blocks for urethroplasty is limited. In 2017, Kalava et al. published a case series describing three patients that underwent pudendal blocks prior to urethroplasty using a trans-perineal approach. None of the three patients required narcotic pain medications while inpatient [21]. In 2019, Jonnavithula et. al. enrolled 30 patients in a small randomized control trial of infraorbital nerve blocks, and showed that the blocks reduced pain scores and facilitated earlier food intake, but they did not assess the impact on opioid use [22]. In 2020, Chua et al. published a single-blinded randomized control trial of liposomal bupivacaine infiltration at the time of hydrodissection for BMG harvest. Twenty-one patients received the liposomal bupivacaine infiltration, while 22 patients received standard hydrodissection (2% xylocaine with epinephrine). Patients had a significant decrease in mean morphine milligram equivalents (MMEs) required on post-operative day one (8.58 mg, *p* = 0.017), however, there were no significant differences on post-operative day two, or among mean pain scores across the two groups [19]. 

Instead of liposomal bupivacaine infiltration, our cohort utilized a BNB of xylocaine, bupivacaine, and dexamethasone. We added dexamethasone because there is evidence it prolongs block efficacy [23,24]. We show that for the post anesthesia care unit (PACU), on a per day basis, and throughout the hospital stay, the number of MMEs needed decreased after implementation of the block, however, despite this trend, the data did not reach statistical significance. As demonstrated in the histograms in Figure 1, the data was significantly skewed, with a majority of patients receiving zero MMEs in the post-operative period. Nevertheless, a slight trend emerged among patients before receiving BNB implantation, needing more MMEs than those receiving the block, however, this again did not reach significance. We expected the analgesic effect of the BNB to last beyond PACU, as blocks are thought to delay the progression to chronic pain and reduce inflammation [8,9,10]. Thus, even after the analgesia wears off, it is thought that nerve blocks may prevent future pain and the need for opioids in the long term. 

Moreover, it is clear from our data that regardless of whether a BNB was performed, a large group of patients did not require any MMEs post-operatively. Byrne et. al. previously reported on a narcotic-free pathway for urethroplasty, which showed no changes in pain scores, despite a significant reduction in opioids prescribed [25]. As urologists continue to practice responsible narcotic stewardship, it appears that for many patients undergoing BMG urethroplasty, there is no need to provide narcotic pain management. 

Our data is limited by its retrospective nature and the limitations that are inherent to retrospective studies. Even though demographic factors between both cohorts are well matched, there may be other differences in patient-related factors or patient care that influenced the results of our study. Specifically, our change in practice patterns in January 2019 to performing BNBs did not occur in a vacuum. By this time, the public consciousness in the United States began to challenge opioid use as the opioid epidemic became more widely publicized. It is thus possible that conscious or subconscious changes in prescriber patterns contributed to the trends observed in our data. 

Moreover, a majority of patients in the study did not require any MMEs post-operatively. Although this potentially provides evidence as to the efficacy of nerve blocks, patients that had a high burden of MME requirements had a large influence over our data set. It is possible patients with high narcotic tolerance were skewing the results, however detailed demographic factors, including exposure to opioids pre-procedure, were not available in this retrospective review.

Finally, we did not see any adverse events from the BNB. There were no reports of severe reactions, including trouble swallowing, difficulty eating, hematomas, infections, or systemic reactions. Thus, there are minimal downsides to performing BNB at the time of BMG harvest.

Despite the limitations in our data set, this study provides evidence that performing buccal nerve blocks intra-operatively is feasible. Our data suggests a possible trend towards lower narcotic requirements for patients receiving a BNB, however it failed to reach statistical significance.

Further research is needed to assess patient-reported pain associated with BNBs, as well as prevention of opioid use in the outpatient setting. In a future randomized trial, we hope to answer these questions, as well as provide more robust data on the effect of the BNB on inpatient narcotic usage. Moreover, future studies should look at the use of non-narcotic pain control, both individually and in combination with nerve blocks. In one such study, Ghiasy et. al. conducted a randomized double-blind placebo-controlled trial of gabapentin vs. placebo among 100 patients, and showed that the gabapentin group had decreased pain scores and MME requirements [26]. 

Going forward, the urologist has clear alternatives for non-narcotic pain management. Given the dangers of opioid prescriptions, both to individual patients and the community at large, every effort should be made to understand the benefit of alternative regimens and transition away from narcotics when possible. The use of BNBs can easily be adopted by other reconstructive urologists.

## Figures and Tables

**Table 1:** Demographic factors for both Non-Buccal Nerve Block and Buccal Nerve Block groups.

		Non-Buccal Nerve Block		Buccal Nerve Block	
		Count	Percent	Count	Percent
	All Patients	37	100%	37	100%
Incision	Penile	20	54.1%	18	48.6%
	Perineal	8	21.6%	12	32.4%
	Penile and Perineal	5	13.5%	7	18.9%
	Female	4	10.8%	-	
Length of Stay	Ambulatory	4	10.8%	6	16.2%
	1 Day	31	83.8%	25	67.6%
	2 Days	2	5.4%	5	13.5%
	3 Days	0	0%	1	2.7%
Race	White	33	89.1%	24	64.8%
	AA	1	2.7%	3	8.1%
	Other	3	8.1%	10	27.0%
Ethnicity	Hispanic	26	70.2%	23	62.1%
	Non-Hispanic	11	29.7%	14	37.8%
		Mean	SD	Mean	SD
Age (Years)		61.48	12.75	59.70	15.37
Intra-operative Stricture Length (cm)		5.64	3.76	5.20	2.66
Intra-operative BMG Area (cm^2^)		7.81	5.86	7.98	5.08

**Table 2:** Mean morphine milligram equivalents (MMEs) for both Non-Buccal Nerve Block and Buccal Nerve Block groups stratified by phase of care. * Mann–Whitney U Test.

	Non-Buccal Nerve Block				Buccal Nerve Block				
	Mean	SD	Median	IQR	Mean	SD	Median	IQR	*p*-Value *
PACU	1.80	2.38	0	0–7.5	1.68	2.98	0.8	0–4.5	0.81
MME/Day	8.78	9.18	8	0–14.5	5.03	6.61	1.6	0–9.1	0.12
Total MME	9.56	10.56	8	0–15	6.5	9.26	1.6	0–10.7	0.20

## Data Availability

Data available on request. The data presented in this study are available on request from the corresponding author. The data are not publicly available due to privacy restrictions.
